# The Air and the Telescope

**Published:** 1885-09

**Authors:** 


### The Air and the Telescope.

The air we breathe is in truth the worst enemy of the astronomer's observations. It is their enemy in two ways. Part of the light which brings its wonderful, evanescent message across inconceivable depths of space, it stops; and when it does not stop, it shatters. And this even when it is most transparent and seemingly still; when mist-veils are withdrawn, and no clouds curtain the sky.

Moreover, the evil grows with the power of the instrument. Atmospheric troubles are magnified neither more nor less than the objects viewed across them. Thus, Lord Rosse's giant reflector posseses —nominally—a magnifying power of 6,000; that is to say, it can reduce the apparent distances of the heavenly bodies to 1-6,000 under their actual amount. The moon, for example, which is in reality separated from the earth's surface by an interval of about 234,000 miles, is shown as if removed only thirty-nine miles. Unfortunately, however, in theory only. Professor Newcomb compares the sight obtained under such circumstances to a glimpse through several yards of running water, and doubts whether our satellite has ever been seen to such advantage as it would be if brought—substantially, not merely optically—within 500 miles of the unassisted eye.—*Popular Science Monthly.*

